# Role and values of Swedish community hospitals through the eyes of general hospital doctors: a qualitative interview study

**DOI:** 10.1136/bmjopen-2026-124543

**Published:** 2026-10-15

**Authors:** Emil San Sebastian Hurtig, Ina Asklund, Margareta Brännström, Mante Hedman

**Affiliations:** 1 Department of Public Health and Clinical Medicine, Umeå universitet Medicinska fakulteten, Umeå, Västerbotten, Sweden; 2 Department of Nursing, Umeå universitet Medicinska fakulteten, Umeå, Västerbotten County, Sweden

**Keywords:** Delivery of Health Care, Integrated, Hospitals, QUALITATIVE RESEARCH, Organisation of health services

## Abstract

**Abstract:**

**Objective:**

To explore how doctors at general hospitals perceive the role and values of community hospitals (CHs) in rural northern Sweden.

**Design:**

Interview study, using qualitative content analysis.

**Setting:**

The study was conducted in Norrbotten and Västerbotten counties in northern Sweden and included three general hospitals.

**Participants:**

Semistructured interviews were conducted during 2025 with nine doctors at general hospitals.

**Results:**

Three main themes were developed: *Offering care close to home*, *Providing safe care far from the hospital* and *Being part of the same organisation*. CHs were seen as important in providing not only standard primary care but also both inpatient and emergency care close to patients’ homes. CHs were also regarded as crucial for patient safety, with experienced staff seen as particularly important to achieve this. Healthcare provision in northern rural Sweden was described as a shared mission with a shared patient population and mutual challenges.

**Conclusion:**

Doctors at general hospitals view CHs as essential in providing healthcare in rural northern Sweden. To provide care safely, CHs are dependent on having both organisational and structural preconditions met.

**STRENGTHS AND LIMITATIONS OF THIS STUDY**

- The interviewer had limited prior experience of the study subject, reducing bias beforehand, which was combined with strong clinical and qualitative expertise among the other researchers. Prior backgrounds within medicine and the studied context bring strength to the analysis while also influencing assumptions.
- Three general hospitals were included, together collaborating with 10 out of the 13 Swedish CHs.
- Although the number of doctors was small, the interviews were considered rich and provided sufficient data for the study.
- Transparency, contextual detail and consistency with prior Swedish research support transferability, though international comparisons require caution.

## Introduction

 Community hospitals (CHs) have been an established component in providing healthcare in northern rural Sweden. Internationally, there is no single definition of a CH nor what services they provide. This varies between and within countries.^1^ Common for many CHs is that they are staffed by general practitioners (GPs) who have responsibility for the inpatients.^2–4^ A scoping review looking at CHs in high-income countries found that most CHs are located in rural areas, although some are situated in urban areas.^1^ The services provided generally reflect local geographical needs and vary substantially.^1^ Internationally, several countries have made efforts to strengthen CHs^2 5^ with the aim, among other objectives, of providing care at an appropriate level and reducing inappropriate use of general hospital beds.^5^ In Sweden, most healthcare is publicly funded, with responsibility shared between the state, the 21 regions and the 290 municipalities. Regions organise and finance primary, secondary and tertiary care, with CHs falling under primary care, while municipalities are responsible for the delivery of nursing care to the elderly and those with functional impairment.^6 7^ With an ageing population, especially prominent in rural Sweden, and an increase in chronic disease prevalence, the healthcare organisations stand before huge challenges.^8–10^ Sweden’s initiative ‘Good Quality and Local Healthcare’ (in Swedish *God och nära vård*) seeks to offer a patient-centred and integrated healthcare with primary care at the centre.^11^

CHs collaborate with the general hospitals, in most instances with the local secondary care general hospital and, when needed, with tertiary care. Within the region, the different levels of care have common electronic health records, with consultations happening over phone or through referrals. CHs also have close collaboration with the local municipalities and their nursing homes.^5^ CH inpatient beds can both facilitate transfer from general hospitals and allow for patients to be admitted directly without transferring patients to a general hospital. Reports show a decrease in general hospital admissions in areas where CHs are available^2^; additionally, there is evidence of more timely and adequate discharge of patients.^12 13^ International studies show that CHs can play an important, although challenging, role in providing secondary and emergency healthcare services in rural areas, where distances to hospitals can be long.^1^ Previous studies support the notion that the Swedish CH model could support the goal of achieving cooperation between healthcare organisations and continuity in care.^4^ Qualitative research on the CHs in Sweden is limited, with only one interview study with CH doctors^8^ and at least one including staff from CHs when analysing policy implementation.^14^ However, the perspectives of general hospital doctors on the CH model have not been studied previously. This group could provide valuable insights into medical, organisational and contextual aspects. The aim of this study is to explore how general hospital doctors in Northern Sweden perceive the role and contribution of CHs in rural healthcare, including their benefits and challenges in collaboration.

## Materials and methods

### Study setting

Currently, there are 13 CHs in Sweden distributed in the regions of Norrbotten, Västerbotten and Jämtland. See figure 1 for an overview of geographical distribution. Together they support almost one-quarter of Sweden’s land area. The municipalities in which Sweden’s CHs are situated are very sparsely populated (0.78 people/km^2^),^4^ with road distances to general hospitals varying between 50 and 230 km. The Swedish CHs are staffed by GPs, nurses, midwives, physiotherapists, occupational therapists, psychologists and more depending on local needs and the decided mission. Beyond their primary care mission and inpatient beds, they can offer basic radiological services, emergency rooms and point-of-care testing. These CHs provide an intermediate level^15^ of care between general hospitals and municipal nursing homes. Patients can be treated for a range of acute conditions and end-of-life care as well as aftercare following general hospital admissions.^4 16^ There have been political discussions of further closures of inpatient beds at several CHs in the region of Västerbotten.^17 18^

**Figure 1:**
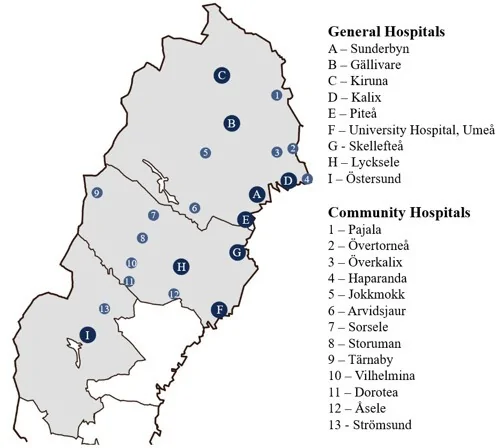
A map of Northern Sweden with regions containing community hospitals (shaded), from top to bottom: Norrbotten, Västerbotten and Jämtland, and the distribution of general hospitals (A to I) and community hospitals (1 to 13) in these regions.

### Design and sampling

This study uses a qualitative exploratory design.^19^ Hospital managers at general hospitals collaborating with CHs were first contacted by MH regarding approval to interview their staff during office hours and to obtain contact details for their doctors. Inclusion criteria were specialist doctors, residents in specialist training or registered medical practitioners with varying experience of collaboration with CHs but with at least 1 year of experience working at that hospital. Subsequently, all provided general hospital doctors (n=30) were invited by ESSH by email, where they received written information about the study, including the purpose of the study as stated in the introduction, that the interviewer ESSH is a medical student and that MH is the responsible researcher for the study. Furthermore, information regarding the management of personal information and a consent form to be filled out before setting up the interview.

### Data collection

Semistructured interviews were conducted over video conferencing by ESSH in Swedish with participating doctors either at their workplace or at their home between February and April of 2025. The interviews were recorded with an external recording device. Open-ended as well as follow-up questions were used, supported by an interview guide created by MH and ESSH to focus discussion on the areas of interest (see online supplemental file 1). Field notes were made after each interview. After the first two interviews, MH and ESSH evaluated the interview guide; no changes were made. No repeat interviews were carried out. The interviews varied between 52 and 87 min. There was no presence of non-participants. The first three interviews were transcribed verbatim by ESSH; the rest were transcribed using the AI software NoScribe (V.0.6)^20^ and were then revised by ESSH. The participants were numbered 1–9 and anonymised by ESSH, and transcribed interviews including ESSHs’ initial reflections were subsequently made available for the research team.

### Data analysis

The transcripts were analysed using qualitative content analysis with an inductive approach,^19 21^ creating codes, grouping them into subcategories and categories and expanding to subthemes and overarching themes with the help of the qualitative data-management software programme MAXQDA V.24.8.0^22^ to systematically sort and code the data. All researchers read all interviews, and one interview was chosen and coded individually by each member and then compared with find common ground. All interviews were subsequently coded and categorised primarily by ESSH and then shared with the rest of the research group allowing for consensus discussion. Quotes from the interviews were selected to illustrate the findings. The participants have not been invited to provide feedback on the transcripts or findings. An example of analysis is provided (figure 2) to better illustrate the process. The study reporting followed the consolidated criteria for reporting qualitative research (COREQ) guidelines.

**Figure 2:**
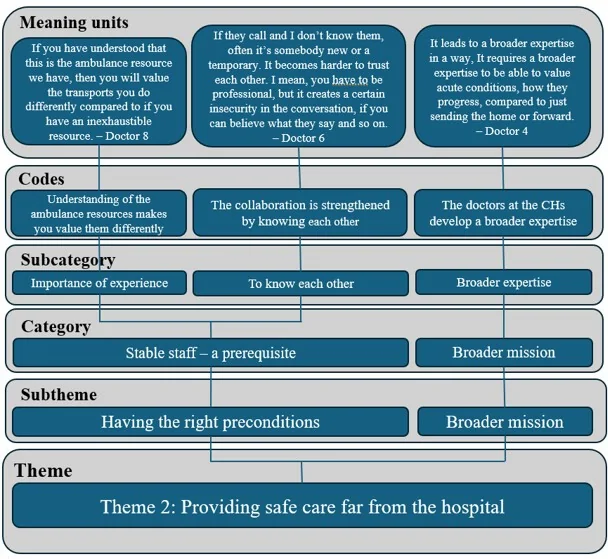
Example of the analysis process, showing the path from meaning unit to theme. CHs, community hospitals.

### Patient and public involvement

Patients and/or public were not involved in the design, conduct, reporting, or dissemination plans of this research.

## Results

### Study participants

Out of the 30 doctors invited to participate, nine accepted within the timeframe of this project and were included in the study. The characteristics of the study participants are summarised in table 1.

**Table 1:** Participants characteristics

Characteristic	Number of participants
Sex	
Men	6
Women	3
Medical specialty	7
Surgical specialty	2
Experience of collaboration with CH (years)	
0–5	2
5–10	2
10–15	2
15–20	1
20+	2
Position	
Resident	3
Specialist	6
County	
Norbotten	4
Västerbotten	5

### Overview of results

Three main themes were developed (see table 2 for an overview), each with their respective subthemes. The first theme, *Offering care close to home*, relates to the role, possibilities and challenges for CHs in providing care close to the patients and their local context. The second theme, *Providing safe care far from the hospital*, concerns the CHs’ mission in providing safe care outside the hospital, the conditions required, and the associated challenges. The third theme, *Being part of the same organisation*, reflects a broader view of healthcare provision in rural Sweden, highlighting a shared mission, common patients and mutual challenges.

**Table 2:** Overview of results, showing themes and subthemes

Theme	Subtheme
Offering care close to home	Providing more care closer to the patient’s home
	Providing care for an ageing population
Providing safe care far from the hospital	Having a broader mission
	Finding the right patients
	Having the right preconditions
Being part of the same organisation	Sharing views
	Treating the same patients
	Allocating limited resources

### Theme 1: offering care close to home

The first theme, *Offering care close to home*, has two subthemes: *Providing more care closer to the patient’s home* concerning how the CHs can provide care closer to the patients. The second subtheme, *Providing care for an ageing population*, describes how CHs can particularly benefit the elderly multimorbid and palliative patients.

#### Providing more care closer to the patient’s home

The participants described the value of having inpatient beds close to the patients. With distances to the general hospital being long, CHs reduce the need for transport and travel for patients. The inpatient beds also increase the treatment possibilities at the CHs. Intravenous treatments, such as intravenous antibiotics for more advanced infections or diuretics for patients with heart failure, were regarded as especially beneficial, as they otherwise would have to be administered at a general hospital. In addition, the continuation of inpatient care closer to home after initial assessment and treatment at the general hospital for more complicated conditions was valued because it allowed for more adequate use of the general hospital’s technical resources.

…when we live in a country with big distances, providing good care close to patients and relatives has a great intrinsic value*.* Doctor 4

The role of CHs in emergency care was highlighted given the long distances, providing patients with a sense of security. On one hand, CHs are able to provide on-site treatments for conditions that do not require extensive technical resources. On the other hand, they can initiate potentially lifesaving treatments earlier for critically ill patients to a greater extent than the ambulance. For example, starting antibiotic and fluid resuscitation for patients with sepsis or administering thrombolysis for myocardial infarctions. The possibility to perform more thorough assessments was described as beneficial, allowing for the CHs to fill a role described as a ‘*filter function*’. CHs can either direct the patient to the appropriate instance of care, considering that different regional hospitals can offer different services, or give suitable care at the CH, often done in collaboration with the doctors at the general hospitals. Serving as primary care centres, CHs allowed for deeper knowledge of patients, reducing unnecessary hospital admissions. Several participants shared the belief that knowledge about the patients, resources and context among CH doctors enables more efficient use of limited ambulance resources, as one doctor described it:

The understanding of there being one ambulance, and if I send that away as a transport, then there is no ambulance*.* Doctor 8

Doctors did not fully agree upon whether CHs provide more outpatient care compared with an ordinary primary care centre. Some thought there were no differences, while those who believed that there were differences attributed them to more resources, broader expertise and long distances to general hospital. By handling a wider range of diagnoses compared with primary care centres in cities, CHs enabled more prompt transfer of outpatients from the general hospital to the CH, thus saving travel for patients. Some doctors also mentioned the ‘*language principle’*, referring to the substantial number of patients who live close to the border with Finland and only speak Finnish. CHs in these areas have more Finnish-speaking staff compared with the general hospital. Therefore, they can communicate in the patients’ native language to a greater extent than possible at the general hospital, which is regarded as beneficial for care.

#### Providing care for an ageing population

One patient group repeatedly mentioned was the multimorbid elderly, a group that particularly benefits from continuity in care provided by the CHs. The opportunity for patients to receive both outpatient and inpatient care from their primary care facility allowed for increased continuity of care. Patients with cognitive impairment were often suggested to benefit from care in a more familiar environment. When discussing the suitability of GPs caring for inpatients, one participant said:

In many cases much better. Here we have a general practitioner who is accustomed in caring for older patients, maybe patients with cognitive impairment (…) While at a larger hospital it is like: Which is the big problem? Yes, it is probably the infection, and you end up at the infection clinic, which is poorly educated in diabetes, heart failure and such things. So, I have big faith in our primary healthcare doctors*.* Doctor 7

Providing palliative care at the CHs was described as both ‘*common’* and ‘*underused possibility’*. CHs were described as providing palliative care of medical quality comparable to that of general hospitals, with palliative patients being highly prioritised and having reserved beds for palliative care. CHs also have greater capabilities than home care services in providing supportive treatments, such as blood transfusions, which would otherwise be provided by general hospitals. A key aspect of providing good palliative care that several doctors emphasised was the clear establishment and adherence to treatment limitations. The doctors mentioned situations when treatment limitations were not followed and patients were sent to the general hospital, sometimes dying in the ambulance or at the hospital far away from family.

### Theme 2: providing safe care far from the hospital

The second theme, *Providing safe care far from the hospital*, has three subthemes. The first subtheme, *Having a broader mission*, relates to the mission of the CHs to provide an intermediate level of care far from the hospital. The second subtheme, *Finding the right patients*, delves into which patients and which do not benefit from care at the CHs. The third subtheme, *Having the right preconditions*, concerns the conditions required to achieve safe care far from the general hospitals.

#### Having a broader mission

The participants agreed that the role, or mission, of the CHs within the healthcare organisation is broader than that of primary healthcare centres, which lack inpatient beds. One doctor described it as:

They have a broad mission really. Partly they are a regular primary health care centre. They should have the same responsibilities and the type of patients as a regular primary health care centre [outpatients]. At the same time, a local emergency clinic and handle acute patients (…). And then they are some sort of local hospital with its beds. So, they have a very broad role and expertise to handle most cases. Doctor 1

CH staff were described as having ‘*an understanding of their increased mission’* and contributing more to the healthcare of their patients compared with primary care providers in cities. Being a doctor in rural areas was described as ‘*easier said than done*’, referring to the challenges of providing care so far from a general hospital and requiring doctors at the CHs to be humble about their mission. To be able to take on this broader role, CH doctors both require and, in many cases, possess greater expertise. Some general hospital doctors expressed higher expectations of what CHs should manage compared with other primary healthcare centres in larger cities.

I mean I expect that they should manage to put casts on simple fractures. While when I work in an urban hospital I would never expect primary care to put on a cast*.* Doctor 9

Several doctors mentioned the need to ensure medical resources close to the patient in order for the CH to fulfil their broader role. An example was X-ray services, when either the technological equipment or knowledgeable staff is lost, so are the diagnostic capabilities. In addition to X-rays, several doctors highlighted the importance of good-quality point-of-care testing with a broad range of analyses. Such resources increase diagnostic and treatment options and help to rule out conditions such as fractures or myocardial infarctions, saving valuable ambulance resources. Other possible areas where participants suggested increasing capabilities were ultrasound and telemetry. One doctor expressed a wish for general hospital specialists to visit CHs to meet their outpatients there, providing the specialist care closer to the patient.

#### Finding the right patients

The CHs were described as ‘*generally patient-safe*’, but they need to be provided with the appropriate conditions. All participants agreed that primary healthcare doctors can be suitable for having these additional areas of responsibility.

…now it sounds like we should have the CHs out of social reasons, having visitors and such. But it’s not only that, it’s also medical safety*.* Doctor 3

Stable staff with good clinical expertise and competence, with adequate resources, were described as necessary to ensure quality and safety. Some doctors emphasised that their response was based on ‘*a feeling’* highlighting a need for further evaluation of working methods and a better understanding of which patient groups are suitable for transfer.

Due to limited diagnostic and treatment options, not all patients benefit from care at CHs. All participants described identifying suitable patients as key in providing safe care at CHs, a process that in many cases is done through active collaboration between the CH doctor and consulting doctor at the general hospital. These patients must be stable while in need of inpatient care for treatment or nursing. Many participants also underlined that in other settings, for example, in cities, these patients would otherwise be admitted to a general hospital.

#### Having the right preconditions

All participants mentioned a stable workforce as key for the CH mission and effective collaboration with the general hospitals. Stable staffing enables personnel to gain experience in the CH’s unique context, including inpatients, emergency care and the treatment of complex or palliative patients. Inpatient work was often mentioned as contributing to the clinical expertise of CH doctors by enhancing their understanding of various diseases, their progression and treatment. This expertise also supports other areas of responsibility, most notably in assessing patients with acute conditions. Understanding of the context and organisation improves consultations and referrals, and a lack of such understanding may ultimately affect patient outcomes.

They have a better understanding for the distances, what’s reasonable, what must be done now and what can wait. Doctor 8

All participants reported good collaboration with the permanent staff at the CHs. Some added that permanent or temporary employment did not matter as long as staff had the necessary competence and understanding of the CH’s context and additional tasks. The participants agreed that a lack of experienced and stable staff created uncertainty for both patients and staff.

…nurses that are stable without big turnover, assistant nurses in the same manner, and doctors in the same manner. That I believe is a precondition if you are going to provide this type of care. If you don’t, everything will be much more uncertain, for both staff and patients, and worse quality too*.* Doctor 4

The participants described a push in the last year to increase the use of digital solutions. Some argued that it leads to increased transfers to the general hospital, while others argued that otherwise all patients would need to be assessed by doctors at the general hospital. However, a lack of trust in and knowledge of digital solutions for remote patient evaluation remains a challenge, particularly among temporary CH staff. This is partly due to insufficient educational efforts and disproportionally affects CHs with a high staff turnover.

… where you have switched to distance consultations. Which, in my experience leads to more patients having to be transferred to the general hospital for assessments. And well, it is not always beneficial for the patients*.* Doctor 9

Several participants reflected on the role of the general hospital in providing support to the CHs. Availability and responsiveness to their colleagues were considered vital in supporting CH doctors. They emphasised that the primary on-call specialist at the general hospital needs a broad clinical expertise. Furthermore, consultations were described as functioning well, with CH doctors having access to several different specialists at the general hospitals, creating a broad support system. Several participants expressed a wish to be more involved in the care of the CH patients.

### Theme 3: being part of the same organisation

The third theme, *Being part of the same organisation*, also has three subthemes. The first, *Sharing views*, is about how general hospitals and CHs share a common goal in providing care in rural Sweden, emphasising the importance of a shared understanding of each other’s contexts. The second subtheme, *Treating the same patients*, portrays the delivery of care as a joint effort. While the third subtheme, *Allocating limited resources*, concerns some of the challenges the delivery of healthcare faces in northern rural Sweden.

#### Sharing views

Several doctors described a shared understanding between the general hospital and the CHs as a foundation for a good collaboration. This includes viewing patient care as a joint responsibility while maintaining a clear division of roles. Such shared understanding is not rigid but a common process that is continuously developed.

We have this basic idea that they are the same patients. They go around, and we help to facilitate for each other. Then we have much better preconditions to help each other out*.* Doctor 4

For example, when new treatments arise or become more available, new directives are needed. One region holds regular meetings between operational managers and medical directors from both general hospitals and the CHs to continuously develop the collaboration. Current challenges include difficulty reaching the doctor responsible for inpatient beds at the CH and knowing bed availability. Doctors from other hospitals without such meetings expressed a wish for something similar. The participants expressed willingness to help from both sides, an experience more prominent in rural areas compared with cities. Long distances were often mentioned as an explanation, and when the collaboration falters,

… then you suddenly feel that this is very far away*.* Doctor 3

The participants described there being a trust in the CHs as well as humbleness towards the challenge CH doctors face when caring for sometimes very sick patients with limited resources far away from the hospital. They acknowledged a need for responsiveness to the CHs’ doctors to contribute to an environment where they can assume responsibility for their patients. An important part of the collaboration is understanding each other’s contexts through their internships and residencies within the local healthcare organisation, whereby doctors gained valued understanding of both sides. Disagreements sometimes arose, with doctors at the CHs, and in relation to multimorbid elderly patients and palliative patients with treatment limitations in place. This was especially evident when an experienced doctor had been on duty during the day, while a temporary on-call doctor was on duty at night and was uncomfortable with the responsibility that these sicker patients entail. This sometimes led to the transfer of patients from CHs to the general hospital during on-call hours, which participants considered could wait until the next day. Generally, there was a wish for more extended on-call staffing and some voiced concerns regarding digital solutions for assessing patients.

It is more during on-call-time when there can be problems. When the on-call doctor is a person who does not dare to take responsibility, not even with advice from us, do this or try that, wait until tomorrow and if it is still not okay then send the patient. But they do not feel sure and send the patient late evening or middle of the night*.* Doctor 7

#### Treating the same patients

The role of the CHs within the healthcare organisation was often discussed in relation to one of the general hospitals’ current challenges: the shortage of hospital beds. The participants had different opinions about the use of inpatient beds at the CHs; some thought there were enough beds while others wished for more. Some participants thought the number of beds was not affected by the general hospital, others described them as increased due to the general hospital using them more to offload patients, surpassing local needs. The inpatient beds at the CHs were described as a ‘*buffer’* for ‘*a hospital under pressure’*, helping to decrease the load on the general hospitals’ inpatient care by freeing up beds at the general hospital, and allowing for faster discharge of patients to CHs. The lack of beds at the general hospitals was also seen as a challenge for the collaboration with CHs, forcing earlier discharges of patients to the CHs and pushing the limits of patient safety as more complex patients were transferred. Fearing that this may undermine the trust between general hospitals and CHs, leading to arbitrary decision-making and consequent frustrations.

But I know there was one CH, maybe several, that introduced some sort of arbitrary cut-off. That they wouldn’t care for patients that had a CRP above 100 or something like that. Which I interpret as a result of that they, they experienced that they previously had received patients with a too high burden of care*.* Doctor 5

Transfer of complex patients too early to CHs can lead to their quick return to the general hospitals, resulting in what one doctor called ‘*ping-pong care’*. To exemplify the loss of CHs, some participants used the summer, a time in which some CHs close their inpatient beds temporarily due to staffing shortages. These consequences include an accumulation of patients with a heavy nursing workload at the general hospitals, as well as earlier discharge of patients to the municipal nursing homes, which in some cases were not prepared to care for those patients. The loss of resources would also lead to fewer opportunities for collaboration, weakening the communication regarding inpatient care.

#### Allocating limited resources

Many participants raised economic issues as a threat to CHs, noting a push towards centralisation and the removal of resources from CHs over the last year. This issue is not unique to CHs but also affects rural general hospitals. However, CHs have generally been affected more due to several reasons, including having smaller financial and workforce buffers, economic compensation for primary care differing from that of general hospitals and a tendency among policymakers to target the smallest actors first. Most doctors described the beds at CHs as cheaper than those at the general hospitals but still relatively expensive due to the low number of beds per CH. They encouraged joint economic solutions with municipalities to reduce costs. Participants raised the question of what the economic consequences of moving resources from CHs to general hospitals would be in the long run.

There are always talks about the economic side of things [regarding areas of responsibility]. This shouldn’t be done at the CH, it should be done at the general hospital, even when it’s not necessary. (…) But maybe it’s cheaper care [provided by the CH] in the long run than putting everything on the general hospital Doctor 1

The participants expressed a wish to retain resources at the CHs, with inpatient beds being particularly important, as they are central to the CHs’ ability to fulfil their mission. Inpatient beds imply greater responsibilities for the CH doctors and must therefore be given the necessary resources to manage. As stated above, a need for experienced staff was described, as a lack of experienced doctors sometimes forces less experienced doctors to take on responsibilities for which they are not equipped. While investments to create long-term conditions to promote staffing in CHs were seen as necessary, a lack of resources reinforced by restrictive financial management was described as the reality, contributing to a stressful climate, increased territorial thinking and worsening of collaboration. Many participants put CHs and the provision of care in rural areas in relation to current Swedish healthcare policy, ‘Good Quality and Local healthcare’. Participants described a discrepancy between proposed policy adjustments and the economic austerity being implemented, which has led to frustration. They expressed the need for politicians to provide CHs with the economic conditions necessary for continued operation.

…if you are going to do the investments that they say they are going to do, there is nothing that speaks towards decreasing the CHs mission /…/ at the same time there is economic austerity which I believe threatens the CH model*.* Doctor 9

## Discussion

In this study, doctors at three general hospitals which collaborate with CHs in Norrbotten and Västerbotten county were interviewed; as a result, three themes were developed: *Offering care close to home*, *Providing safe care far from the hospital* and *Being part of the same organisation*. The participants regarded the CHs’ role in providing healthcare in northern rural Sweden as significant, offering an intermediate level of care^10^ closer to patients’ homes and providing relief to general hospitals under pressure. Strong collaboration with general hospitals, along with a stable and competent workforce, were identified as key prerequisites for this role. Similar models to the Swedish CHs exist worldwide, primarily in rural areas, and rely on generalist doctors.^3^ The research about the Swedish CHs is limited and has mainly focused directly on the CH and specific interventions^4 16^ or them being part of local case studies.^14^ The results of this study contribute a perspective from collaborating doctors outside the CH, yet still part of the same healthcare organisation and similar context. In the following sections, several aspects of the results will be discussed, including the provision of close care, the importance of inpatient beds and continuity of staff in relation to international literature. Furthermore, a brief discussion of the findings in regard to current Swedish healthcare policy and resource use is also provided. The importance of providing care close to the patients, especially for elderly and multimorbid patients, is not a unique finding. The interviewed doctors placed value in both contextual closeness, as described previously as important by patients,^23^ and geographical closeness, placing value in convenience for patients as well as medical safety. The inpatient beds were often mentioned by the participants as central to the CH, as they differentiate them from ordinary primary care centres.^2–4^ The importance of inpatient beds is recurring through the three themes in this article. They play an important role in the CHs’ ability to offer more aspects of care close to the patients. The possibility to admit patients facilitates the management of many of the conditions from which the elderly suffer, such as infections and chronic diseases such as chronic obstructive pulmonary disease and heart failure.^4 16^ The beds also add to the CHs’ ability to provide end-of-life care, often serving as substitute hospices.^24^ The participants in our interviews argued for the importance of continuity in staff and clear treatment limitations for delivery of successful palliative care, which is in line with previous research.^25 26^ The Swedish CH doctors have previously argued that CHs are better suited than general hospitals for the care of palliative patients.^16^ The continuity in care which the CHs provide through their inpatient beds and outpatient care, delivered by the same GPs, was seen as an important feature of the CH by our participants. The continuity was perceived to contribute also to the clinical competence and creation of a more stimulating working environment, as also reported by the Swedish CH doctors.^16^ For the elderly patients with multimorbidity, continuity of care was especially important, promoting a more person-centred approach, providing an appropriate level of care and reducing low-value interventions.^27 28^ The admission of patients to the CH or general hospital was often done in dialogue between doctors at the CH and the hospital. The Swedish CH doctors reported that besides the differences in resources mentioned by the interviewed doctors in this article, the clinical experience and skill of the individual CH doctor could also influence which patients would be admitted.^16^ Although doctors in this study considered many aspects of providing safe care, the continuity of staff at both CHs and general hospitals was arguably among the most important, as it has been shown to influence patient outcomes.^29^ CH care was generally considered safe by general hospital doctors, and understanding which patients will benefit from admission to CH or general hospital was seen as a continuous collaborative process. CH doctors have previously described having to continuously assess risk, among other things, and manage the use of limited ambulance resources.^16 30^ An issue often brought up also by the participants in our study, with them arguing for keeping both technological and human resources close to the patient to reduce the need for transportation. Sweden’s ‘Good Quality and Local healthcare’ (in Swedish *God och nära* vård) reform^11^ aims for patient-centred and integrated healthcare prioritising efficiency and individual needs with a focus on primary care and increased continuity.^31^ The results from our interviews strengthen the idea that CHs arguably may play an important role in achieving the aims of this reform in Northern Sweden,^16^ however constrained by limited financial and human resources.^11 14 32^ In the ‘Good Quality and Local Care’ Act, primary healthcare is described as the ‘hub’ for the patient’s care and is viewed as central in the integration of services.^11^ To better comprehend the role of CHs in providing integrated care, the results from these interviews, as well as previous research,^14 16^ can be understood through different aspects of integrated care.^33 34^ The CHs provide an intermediate level of care offering both emergency care and inpatient beds close to the patients. According to the participants, this position is vital for maintaining safe care in northern rural Sweden. Thanks to the inpatient beds and emergency care services, the CHs are allowed to integrate services on one hand vertically, throughout different levels of care.^22^ Serving as a stepping stone between the general hospital and municipal care, as well as for emergency care, providing advanced treatment and diagnostic resources closer to the patient before transferring to the general hospital. The CH also becomes an alternative for care that otherwise would have to be given at the general hospital, thereby ‘decreasing pressure’ on the general hospital. As Sweden aims to become among the best countries in the world for the use of digital solutions in healthcare,^35^ doctors from the general hospitals had their reservations. Digital solutions are being implemented without adequate training and knowledge retention, a problem that is especially notable at CHs with high staff turnover or a high degree of medical locums. An issue reported earlier by both CH doctors and managers from the same studied context includes the lack of IT support and insufficient adaptation of digital solutions.^14 16^ All participants described various aspects of how CHs allow for more efficient usage of resources. Still the Swedish CH model is described as hollowed out, where economic arguments have been paramount. There was a fear that further budget cuts would lead to increased financial management and tendencies to limit responsibility from both CHs and general hospitals. Many participants thought of the CHs as having cheaper inpatient beds compared with the general hospital, a notion shared with the doctors at the Swedish CHs,^16^ but not yet verified in any Swedish cost-effectiveness study. Internationally, British^36 37^ and Norwegian^38–41^ research suggests that CHs can excel or match general hospitals in health outcomes, readmissions, independence and cost-effectiveness. Several of the participants shared the experience that CHs reduce the amount of admissions to the general hospital due to being admitted to the CHs, a suggestion supported by Norwegian studies.^41 42^ The interviewed doctors argued against operational cuts and centralisation to fewer CHs due to budget restrictions and instead supported long-term financial investments to secure stable staff, inpatient beds and expanding the diagnostic capabilities. The consequences of centralisation are not limited to CHs but also general hospitals, an issue not unique to Sweden.^32^

### Strengths and limitations

The interviewer, ESSH, a male medical student at Umeå University, had no previous experience of CHs outside of the medical programme, which may reduce bias but still provided a basic medical and contextual understanding. Contextual experience came from MH, who has a background as a clinician at a CH and researcher in this field as well as qualitative research. Further strength to the analysis was brought by IA and MB who contributed with expertise in qualitative methods. The authors are native Swedes with backgrounds in medicine and nursing with experience of the studied healthcare system, which may have influenced both data collection and analysis by leading to assumptions about certain norms and practices and potentially overlooking alternative viewpoints. This study includes three general hospitals, which collaborate with a majority (10/13) of Swedish CHs. The participants had diverse levels of clinical experience providing a range of perspectives, although most were doctors with an internal medicine specialty. In accordance with the principles of qualitative content analysis,^21^ data collection was considered sufficient when the material provided rich and varied descriptions relevant to the study aim. Data saturation was assessed during the analysis, and the final interviews did not generate substantially new codes or categories. Despite the relatively small sample, the data were considered sufficiently rich to illuminate different perspectives on the role and value of CHs in rural northern Sweden. We aimed for transparency in the presentation of the analysis process, exemplified in figure 2 and in the citations. In terms of transferability, the paper includes a short description of the Swedish healthcare system as well as a detailed description of the study setting, allowing readers to draw comparisons to their own contexts.^43^ Studies also targeting the Swedish CH model directly or indirectly show similarity in many of the findings, strengthening these results.^14 16 44^ When comparing with international research, both size and services of CHs vary to meet local needs, as well as organisation and funding, aspects that must be considered when comparing to research from outside of Sweden.

## Conclusion

General hospital doctors perceive CHs as important components of rural healthcare in Northern Sweden, given the specific geographical and organisational context. CHs are described as functioning as an intermediate level of care, supporting the use of general hospital resources while maintaining local responsibility for appropriate patient groups. By providing inpatient and emergency care close to patients’ homes, CHs are perceived to enhance accessibility, continuity and context-adapted care, particularly for multimorbid older adults and patients with palliative care needs. The capacity to provide safe care appears to depend on appropriate organisational and structural preconditions, including stable and experienced staffing, adequate diagnostic resources, clear patient selection processes and structured collaboration with general hospitals. Further research is needed to explore patient safety outcomes and the cost-effectiveness of this intermediate care model.

## Supplementary material

10.1136/bmjopen-2026-124543 online supplemental file 1

## Data Availability

Data are available upon reasonable request.

## References

[1] Winpenny EM, Corbett J, Miani C (2016). Community Hospitals in Selected High Income Countries: A Scoping Review of Approaches and Models. Int J Integr Care.

[2] Heaney D, Black C, O’donnell CA (2006). Community hospitals--the place of local service provision in a modernising NHS: an integrative thematic literature review. BMC Public Health.

[3] Community hospitals-preparing for the future (1990). A report from the Liaison Group of the Royal College of General Practitioners and Associations of General Practitioner Community Hospitals. Occas Pap R Coll Gen Pract.

[4] Hedman M, Boman K, Brännström M (2021). Clinical profile of rural community hospital inpatients in Sweden - a register study. Scand J Prim Health Care.

[5] Tucker H (2013). Discovering integrated care in community hospitals. Journal of Integrated Care.

[6] (2017). Hälso- och sjukvårdslag (SFS 2017:30). https://www.riksdagen.se/sv/dokument-och-lagar/dokument/svensk-forfattningssamling/halso-och-sjukvardslag-201730_sfs-2017-30/.

[7] Ludvigsson JF, Bergman D, Lundgren CI (2025). The healthcare system in Sweden. Eur J Epidemiol.

[8] Kingston A, Robinson L, Booth H (2018). Projections of multi-morbidity in the older population in England to 2035: estimates from the Population Ageing and Care Simulation (PACSim) model. Age Ageing.

[9] Socialstyrelsen (2014). Grund och struktur för lägesrapportering om kroniska sjukdomar. https://www.socialstyrelsen.se/globalassets/sharepoint-dokument/artikelkatalog/ovrigt/2014-9-40.pdf.

[10] Eurostat (2025). Demography report 2020 - population and demography: Sweden. https://ec.europa.eu/eurostat/web/population-demography/demography-population-stock-balance/publications/demography-report.

[11] Sveriges kommun och Regioner (2023). Överenskommelse - God och nära vård 2024.

[12] Dahl U, Steinsbekk A, Jenssen S (2014). Hospital discharge of elderly patients to primary health care, with and without an intermediate care hospital - a qualitative study of health professionals’ experiences. Int J Integr Care.

[13] Garåsen H, Windspoll R, Johnsen R (2007). Intermediate care at a community hospital as an alternative to prolonged general hospital care for elderly patients: a randomised controlled trial. BMC Public Health.

[14] Blåhed H, Jonsson F, Hurtig AK (2024). In-Between Policy Vision and Practical Realities of Primary Healthcare: A Case Study in Rural Northern Sweden. Int J Health Policy Manag.

[15] Melis RJF, Olde Rikkert MGM, Parker SG (2004). What is intermediate care?. BMJ.

[16] Hedman M, Wennberg P, Sjöström M (2025). Role of general practitioner-led rural community hospitals in Sweden: a qualitative study. BMJ Open.

[17] (2025). Västerbotten går vidare med nedskärningar: »Sänkta ambitioner. https://lakartidningen.se/nyheter/vasterbotten-gar-vidare-med-nedskarningar-sankta-ambitioner/.

[18] SVT Nyheter (2025). Oro i malå kring nytt sparförslag:”Är bedrövligt”. https://www.svt.se/nyheter/lokalt/vasterbotten/oro-i-mala-kring-nytt-sparforslag-ar-bedrovligt.

[19] Graneheim UH, Lundman B (2004). Qualitative content analysis in nursing research: concepts, procedures and measures to achieve trustworthiness. Nurse Educ Today.

[20] Dröge K (2024). NoScribe. AI-powered audio transcription. https://github.com/kaixxx/noScribe.

[21] Graneheim UH, Lindgren BM, Lundman B (2017). Methodological challenges in qualitative content analysis: A discussion paper. Nurse Educ Today.

[22] Software V https://www.maxqda.com/.

[23] Green J, Forster A, Young J (2008). Older people’s care experience in community and general hospitals: a comparative study. Nurs Older People.

[24] Rainsford S, MacLeod RD, Glasgow NJ (2016). Place of death in rural palliative care: A systematic review. Palliat Med.

[25] Saultz JW, Albedaiwi W (2004). Interpersonal continuity of care and patient satisfaction: a critical review. Ann Fam Med.

[26] International Association for Hospice and Palliative Care (2025). Principles of palliative care: guidelines and suggestions for those starting a hospice/palliative care service. https://iahpc.org/resources/publications/getting-started/principles-of-palliative-care/.

[27] Romano MJ, Segal JB, Pollack CE (2015). The Association Between Continuity of Care and the Overuse of Medical Procedures. JAMA Intern Med.

[28] Barker I, Steventon A, Deeny SR (2017). Association between continuity of care in general practice and hospital admissions for ambulatory care sensitive conditions: cross sectional study of routinely collected, person level data. BMJ.

[29] Prior A, Rasmussen LA, Virgilsen LF (2025). Continuity of care in general practice and patient outcomes in Denmark: a population-based cohort study. *The Lancet Primary Care*.

[30] Hedman M, Doolan-Noble F, Stokes T (2022). Doctors’ experiences of providing care in rural hospitals in Southern New Zealand: a qualitative study. BMJ Open.

[31] Socialstyrelsen (2025). God och nära vård. https://www.socialstyrelsen.se/kunskapsstod-och-regler/omraden/god-och-nara-vard/.

[32] Vaughan L, Edwards N (2020). The problems of smaller, rural and remote hospitals: Separating facts from fiction. *Future Healthc J*.

[33] Valentijn PP, Schepman SM, Opheij W (2025). Understanding integrated care: a comprehensive conceptual framework based on the integrative functions of primary care. *Int J Integr Care*.

[34] World Health Organization (2016). Framework on integrated, people-centred health services.

[35] E-hälsomyndigheten (2025). Vision e-hälsa. https://www.ehalsomyndigheten.se/om-oss/regeringsuppdrag/vision-e-halsa-2025/.

[36] Coast J, Inglis A, Frankel S (1996). Alternatives to hospital care: what are they and who should decide?. BMJ.

[37] O’Reilly J, Lowson K, Green J (2008). Post-acute care for older people in community hospitals-a cost-effectiveness analysis within a multi-centre randomised controlled trial. Age Ageing.

[38] Aaraas I (1995). The Finnmark general practitioner hospital study. Patient characteristics, patient flow and alternative care level. Scand J Prim Health Care.

[39] Dahl U, Steinsbekk A, Johnsen R (2015). Effectiveness of an intermediate care hospital on readmissions, mortality, activities of daily living and use of health care services among hospitalized adults aged 60 years and older-a controlled observational study. BMC Health Serv Res.

[40] Aaraas I, Søråsdekkan H, Kristiansen IS (1997). Are general practitioner hospitals cost-saving? Evidence from a rural area of Norway. Fam Pract.

[41] Aaraas I, Førde OH, Kristiansen IS (1998). Do general practitioner hospitals reduce the utilisation of general hospital beds? Evidence from Finnmark county in North Norway. J Epidemiol Community Health.

[42] Hagen TP, Tjerbo T (2023). The Causal Effect of Community Hospitals on General Hospital Admissions. Evaluation of a Natural Experiment Using Register Data. Int J Integr Care.

[43] Shenton AK (2004). Strategies for ensuring trustworthiness in qualitative research projects. *EFI*.

[44] Ärlebrant L, Dubois H, Creutzfeldt J (2024). Emergency care via video consultation: interviews on patient experiences from rural community hospitals in Northern Sweden. Int J Emerg Med.

