# Supplementary material for: Role and values of Swedish community hospitals through the eyes of general hospital doctors: a qualitative interview study
Source: BMJ Open. 2026 Oct 15;16(10):e124543. doi: 10.1136/bmjopen-2026-124543 (PMC13691799; doi:10.1136/bmjopen-2026-124543)
Supplement: Supplementary file 1 [file bmjopen-16-10-s001.pdf]

Bilaga 3

Intervjuguide – individuella intervjuer med sjukhusläkare om deras erfarenheter att jobba gentemot sjukstugor/hälsocentraler med OBS-avdelning

Börja med att hälsa välkommen. Påminn om syftet med studien, att deltagande är frivilligt och att personen när som helst kan avbryta sin utan att behöva uppge skäl. Be om tillåtelse att spela in intervjun och förklara varför det är viktigt (för att ha bättre möjligheter att analysera). Försäkra att konfidentialitet och integritet kan garanteras. Fråga om möjlighet att återkomma till forskningspersonen efter intervjun för komplettering om det framkommer oklarheter eller om nya frågeställningar dyker upp under studiens gång.

Etikprövningsmyndigheten
2024-07990-01-679992
2024-12-02

Inledning

1. Kan du beskriva hur du jobbar gentemot sjukstugorna i din roll som läkare? (ex: möter inremitterade patienter från sjukstuga på akutmottagning eller planerad mottagning, beslutar om eller verkställer utskrivning av patienter för fortsatt vård vid sjukstuga, bidrar med rådgivning angående patienter inneliggande vid sjukstuga etc)

2. Med vilka yrkesgrupper och på vilket sätt kommunicerar du med sjukstugans personal? (Läkare, sjuksköterskor på vårdavdelning, sjuksköterska under jourtid)

Sjukstugornas roll

3. Utifrån din erfarenhet, vilken roll har sjukstugorna idag? (inom sjukvårdsorganisationen, för patienten och för närsamhället - dessa perspektiv aktuella för alla frågeställningar nedan)
   a. Sjukstugornas vårdavdelning
      i. Vilka patientgrupper lämpliga?
      ii. Varför dessa patienter på sjukstuga och inte på sjukhus? (Berätta gärna med exempel)
   b. Sjukstugornas akutmedicinska åtagande
      i. (Ambulansen stannar vanligtvis vid sjukstuga med patienter som på annat håll skulle köras till ett sjukhus akutmottagning)
   c. Skiljer sig sjukstugornas roll jämfört med ”vanliga” hälsocentralers när det gäller öppenvården/primärvården? (kan du beskriva?)

4. Från dina erfarenheter som läkare på sjukhus, beskriv dina tankar kring sjukstugornas arbete gällande: (Beskriv gärna situationer du själv upplevt)
   a. Akutsjukvård?
   b. Rehabilitering på avdelning?
   c. Palliativ vård på avdelning?
   d. Inneliggande patienter generellt?
   e. Övriga arbetsområden?

5. Vilka utmaningar ser du att sjukstugorna har för att klara av sitt arbete?
    a. Beskriv vad du tycker fungerar bra och vad hade kunnat förbättras? Hur?
    b. Finns det någon verksamhet som sjukstugorna inte bedriver, och som du hade velat se dem göra?

Samarbete

6. Hur skulle du beskriva samarbetet runt gemensamma patienter mellan sjukhuset och sjukstugorna? (nedan olika vanliga samarbetssituationer – beskriv gärna konkreta situationer, positiva eller negativa)
    a. Överflyttningar av patienter mellan sjukhus och sjukstuga (båda vägarna)
    b. Diskussioner kring vårdnivå – inläggning sjukhus vs sjukstuga
    c. Diskussioner kring utredning och behandling av inneliggande patienter på sjukstugan.
    d. Uppföljningar i primärvård efter vårdtillfälle

7. Ifall du har erfarenhet av det: skiljer det sig samarbetet med sjukstugor jämfört med samarbete mellan sjukhus och vanlig hälsocentral utan vårdavdelning? I så fall, beskriv hur?

Etikprövningsmyndigheten
2024-07990-01-679992
2024-12-02

8. Från ditt perspektiv, upplever du att sjukstugorna uppfyller sina arbetsuppgifter på ett patientsäkert sätt?
    a. (Hur ser du på att distriktsläkare sköter inneliggande sådana akutpatienter som på annat håll sköts av sjukhusläkare på sjukhus. Dvs äldre multisjuka med hjärtsvikt, lunginflammation mm)

9. Vilka faktorer tror du stärker samt försvagar samarbetet mellan sjukhus och sjukstugor?
    a. Har du exempel på egna erfarenheter för att beskriva detta?

Avslutning

10. Har du något mer du vill dela med er av/lägga till? Något som vi ännu inte berört/tagit upp?

11. Vad tycker du är det viktigaste som kommit fram i det här samtalet?

Tacka och ge möjlighet för deltagarna att ställa frågor och komma med synpunkter.

Interview guide (translated from Swedish to English)

Interview guide – Individual interviews with general hospital doctors about their experiences in working towards Community Hospitals

Start with greeting them welcome. Remind them of the purpose of the study, that participation is voluntary and that they can whenever terminate the interview without giving a reason. Ask for permission to record the interview and explain why it is important (to better analyse later). Assure that confidentiality and integrity can be guaranteed. Ask about the possibility to maybe contact them later for completion if new questions rise during the process of the study.

Introduction

1. Can you describe how you work towards the community hospitals as a doctor?
2. With which professional groups and in which way do you communicate with the staff at the Community Hospital

The role of Community Hospitals

3. From your experience, which role does Community hospitals have today?
    a. The inpatient ward?
        i. Which patient groups?
        ii. Why should these patients be at the community hospital and not general hospital?
    b. Their work regarding emergency medicine
    c. Do community hospitals differ from "normal" primary care centres when it comes to outpatient care?
4. From your experience as a doctor at a general hospital, describe your thought about the work of Community Hospitals regarding:
    a. Emergency care?
    b. Inpatient rehabilitation?
    c. Inpatient palliative care?
    d. Inpatients generally?
    e. Other areas of work?
5. Which challenges do you see for the Community Hospitals to achieve their work?
    a. What do you think works, and what do you think could be improved? How?
    b. Are there any activities that community hospitals do not perform, that you would like them to?

Collaboration

6. How would you describe the collaboration regarding common patients, between the general hospitals and Community Hospitals?
    a. Transfer of patients from general hospitals to community hospital (both ways)
    b. Discussion about appropriate level of care – admitting at general och community hospital

    c. Discussions regarding further examination and treatment of inpatients at the community hospital
    d. Follow up at primary care after a hospital stay.
7. If you have experience, does the collaboration with Community Hospitals differ compared to Primary care centeres without inpatients? If so, how?
8. From your perspective, do you perceive that Community Hsopitals perform their tasks in a patient safe way?
    a. (How do you view that a primary care doctor has responsibility over inpatients, which in other parts would be cared for at a general hospital. Eg. Multimorbid elderly with heart failure, pneumonia etc).
9. Which factors do you think strengthen and weaken the collaboration between general hospitals and community hospitals?

Ending

10. Do you have something more you would like to share/add? Something we haven't brought up today?
11. What do you think is the most important thing which come out of this conversation?

Thank them and give the possibility for the participant to ask questions and come with comments.
