# Supplementary material for: Role and values of Swedish community hospitals through the eyes of general hospital doctors: a qualitative interview study
Source: BMJ Open. 2026 Oct 15;16(10):e124543. doi: 10.1136/bmjopen-2026-124543 (PMC13691799; doi:10.1136/bmjopen-2026-124543)
Supplement: Supplementary file 2 [file bmjopen-2026-124543.reviewer_comments.pdf]

PEER REVIEW HISTORY

BMJ Open publishes all reviews undertaken for accepted manuscripts. Reviewers are asked to complete a checklist review form and are provided with free text boxes to elaborate on their assessment. These free text comments are reproduced below.

ARTICLE DETAILS

Title (Provisional)

Role and Values of Swedish Community Hospitals Through the Eyes of General Hospital Doctors - A Qualitative Interview Study

Authors

San Sebastian Hurtig, Emil; Asklund, Ina; Brännström, Margareta; Hedman, Mante

---

VERSION 1 - REVIEW

**Reviewer**	**1**
**Name**	**Schmiedhofer, Martina**
**Affiliation**	**Charité Universitätsmedizin Berlin, Emergency and Acute Medicine**
**Date**	**30-Jul-2026**
**COI**	**None**

Thank you very much for the invitation to review this manuscript, which deals with the inpatient care of patients in remote rural areas of Sweden. The concept of community hospitals is convincing for patients whose current medical needs fall between home care or nursing care and inpatient hospital care. Given the vast distances in geographically sparsely populated areas, CHs offer numerous advantages, which in the manuscript is examined from the perspective of doctors working in general hospitals. The subject of this study complements an article published in 2025 from the same region, which I also had the opportunity of reviewing. The findings of the earlier article, which examined the perspectives of general practitioners working in CHs – also obtained through qualitative research – present many similarities with the results of the manuscript at hand. The joint findings from different perspectives underscore the importance of the CH concept.

Healthcare systems that overcome the segregation between inpatient and outpatient care are highly valuable. As mentioned in the introduction of the manuscript, economic numbers show not only an improved patient centred-care, but also a cost-reduction (which is partly negatively connotated as an incentive to implement CH). The core of this topic – the implementation and functioning of CH – would therefore be worth analysing from a health economics perspective or examining from the perspective of organisational health service

research. Nethertheless, the views of physicians working in general hospitals are important for assessing the quality of the care provided by CH and the benefits to patients. In order to fully understand the findings and place them correctly within their organisational context – particularly for non-swedish readers – some further information is required. For example, topic 3, 'Being in the same organisation', cannot be understood without clarification of the term 'organisation': Are CH and general hospitals part of the same administrative level, do they share a formal structure, funding from the same budget,do they follow guidelines for the referral of patients or the exchange of information, or is it merely a matter of communication based on personal and professional commitment? Further, one quote states that CH should have „the same responsibilities and the type of patients' as "other primary healthcare centres"? What is meant by 'other primary healthcare centres?' This statement is also not understandable without the knowledge of more organisational details. As in the article published in 2025, some of the quotations assume that financial constraints lead to the implementation of CH, as the costs are lower than those of general hospitals. Also, reduced budgets threatening CH resources are mentioned in the current article, whereby it remains unclear what will be happening to the patients in need: are they admitted to (the more expensive) general hospital or lacking the care they need? Again, without the financial context, the meaning of the statements remains vague, as it cannot be distinguished between 'the truth' (e.g. economic figures, cuts to hospital beds, replacement by CH) and the subjective of perception of interviewees, which is the subject of qualitative research.

It is therefore recommended that the manuscript is revised from the perspective of a reader who is unfamiliar with the Swedish healthcare system. In this context, it might be useful to focus on the subjective perception of the quality of care, including any barriers. Furthermore, the manuscript would benefit if the results were related to some of the findings from the earlier publication, as the perspectives of physicians working in CH and those working in general hospitals either complement each other well or highlight structural differences, including existing problems.

The study addresses the care needs of a growing patient group by providing a suitable supply of care, which can serve as a model for other countries, not only in remote regions.

VERSION 1 - AUTHOR RESPONSE

<u>**Reviewers comments to the Authors:**</u>

In order to fully understand the findings and place them correctly within their organisational context – particularly for non-swedish readers – some further information is required. For example, topic 3, 'Being in the same organisation', cannot be understood without clarification of the term 'organisation': Are CH and general hospitals part of the same administrative level, do they share a formal structure, funding from the same budget, do they follow guidelines for the referral of patients or

the exchange of information, or is it merely a matter of communication based on personal and professional commitment?

We thank the reviewer for this constructive comment. We have revised the introduction (page 4) to further provide the reader with an understanding of the organisational structure between CHs and general hospitals. It reads now:

> *In Sweden most healthcare is publicly funded, with responsibility shared between the state, the 21 regions and the 290 municipalities. Regions organise and finance primary, secondary and tertiary care, with CHs falling under primary care~~including CHs~~, while municipalities are responsible of the delivery of nursing care to elderly and those with functional impairment (6,7). With an aging population, especially prominent in rural Sweden, and increase in chronic disease prevalence the healthcare organisations stand before huge challenges (8–10). Sweden's initiative "Good Quality and Local Healthcare" (in Swe. God och nära vård) seeks to offer a patient-centred and integrated healthcare with primary care at the centre (11).*
>
> *CHs collaborate with the general hospitals, in most instances with the local secondary care general hospital and when needed with tertiary care. Within the regions the different levels of care have common electronic health records, with consultations happening over phone or through referrals. CHs also have close collaboration with the local municipalities and their nursing homes (5). ~~both primary and secondary care as well as other health care services such as nursing homes (5).~~ CHs inpatient beds can both facilitate transfer from general hospitals and allow for patients to be admitted directly without transferring patients to a general hospital. Reports show a decrease in general hospital admissions in areas where CHs are available (2), additionally, there is evidence of more timely and adequate discharge of patients (12,13).*

Further, one quote states that CH should have „the same responsibilities and the type of patients' as "other primary healthcare centres"? What is meant by 'other primary healthcare centres?' This statement is also not understandable without the knowledge of more organisational details.

Thank you for this comment. The participants often referred to primary care centres which weren't CHs as "other primary healthcare centres", meaning that they lacked inpatient beds. This section (page 13) has been revised to hopefully be more clear. It now reads:

> *The participants agreed that the role, or mission, of the CHs within the healthcare organisation is broader~~greater~~ than that of ~~other~~ primary healthcare centres wich lacks inpatient beds. One doctor described it as:*
>
> *"They have a broad mission really. Partly they are a regular primary health care centre. They should have the same responsibilities and the type of patients as a regular primary health care centre [outpatients]. At the same time, a local emergency clinic and handle acute patients (…). And then they are some sort of local hospital with its beds. So, they have a very broad role and expertise to handle most cases" – Doctor 1*

As in the article published in 2025, some of the quotations assume that financial constraints lead to the implementation of CH, as the costs are lower than those of general hospitals.

Thank you for this comment. As you say, we show in the results under theme 3 (page 21) that the doctors at general hospitals generally believe that care at the CHs can be cheaper or better use of resources compared to having all patients at the general hospitals. Therefore, in the discussion we claim that the doctors at the general hospital and CHs (article from 2025) generally agree that CHs can provider care at a lower cost than the general hospital for adequate patient populations. Several participants in this study therefore expressed reluctancy in different ways towards the closure of CHs and/or proposed budget cuts by the regional governments. No participant in this study expressed that the financial constraints lead to the implementation of more CHs, therefore no change to the manuscript has been made.

Also, reduced budgets threatening CH resources are mentioned in the current article, whereby it remains unclear what will be happening to the patients in need: are they admitted to (the more expensive) general hospital or lacking the care they need?

Thank you for this comment as it highlights an important issue. Some doctors provided their experience of this issue. As stated in the results under Theme 3 (page 19-20) during staffing shortages, i.e the summers, some CHs to close for a limited time as they are not able to provide their services. This results in more inpatients at the general hospital and/or admitted to another CHs. After reviewing it has now hopefully been made clearer, it reads now:

> *To exemplify the loss of CHs some participants used the summer, a time in which some CHs close their inpatient beds temporally due to staffing shortage. ~~Moreover, some doctors described the consequences when CHs close their inpatient beds due to staffing shortages, for example in the summer. These~~ These consequences include an accumulation of patients with a heavy nursing workload at the general hospitals, as well as earlier discharge of patients to the municipal nursing homes, which in some cases were not fully prepared to care for those patients.*

Again, without the financial context, the meaning of the statements remains vague, as it cannot be distinguished between 'the truth' (e.g. economic figures, cuts to hospital beds, replacement by CH) and the subjective of perception of interviewees, which is the subject of qualitative research. It is therefore recommended that the manuscript is revised from the perspective of a reader who is unfamiliar with the Swedish healthcare system. In this context, it might be useful to focus on the subjective perception of the quality of care, including any barriers.

Thank you for this comment making an important point. While many of the participants talked about the financial context, our interview guide did not include any direct questions regarding this. However it was often mentioned when asked either about the challenges CHs face or questions regarding collaboration. The entire results section (page 9-22) consists of our analysis of the interviews through qualitative content analysis as well as quotations by the participants. Therefore, the results should be understood as the subjective thoughts based on the experiences of the participants. There is no Swedish cost-effectiveness study of CHs, making it difficult to translate the experience of the participants into objective data. Nonetheless there are international studies which has looked at this. The section of the discussion (page 26) has now been revised to hopefully make this clearer. It reads now:

Many participants thought of~~described~~ the CHs as having cheaper inpatient beds compared to the general hospital, a notion shared with the doctors at the Swedish CHs (16), but not yet verified in any Swedish cost-effectiveness study. Internationally, British (36,37) and Norwegian (38–41) research suggest that CHs can excel or match general hospitals in health outcomes, readmissions, independence and cost-effectiveness. Several of the participants shared the experience that CHs reduce the amount of admissions to the general hospital due to being admitted to the CH, a suggestion supported by Norwegian studies *(41,42). ~~suggest CHs reduce general hospital admissions, an experience shared by many of this study's participants.~~*

Furthermore, the manuscript would benefit if the results were related to some of the findings from the earlier publication, as the perspectives of physicians working in CH and those working in general hospitals either complement each other well or highlight structural differences, including existing problems.

Thank you for this comment. The authors agree that this is an interesting comparison as both studies have been performed in the same study setting within different parts of the organisation. The forementioned article (reference number 16: Hedman M, Wennberg P, Sjöström M, Brännström M. Role of general practitioner-led rural community hospitals in Sweden: a qualitative study. BMJ Open 2025 Feb) is referenced at several instances throughout the discussion. The discussion has been revised to better show similarities and differences between the two studies. The following changes have been made:

Page 23:

~~In a previous study,~~ The Swedish CH doctors have previously argued that CHs can be~~are~~ better suited than general hospitals for the care of palliative patients (16).

Page 23-24:

The continuity in care which the CHs provide through their inpatient beds and outpatient care, delivered by the same general practitioners, was seen as an important feature of the CH by our participtans. The continuity was perceived to contribute also

to the clinical competence and creation of a more stimulating working environment, as also reported by the Swedish CH doctors (16).

Page 24:

The admission of patients to the CH or general hospital was often done in dialogue between doctors at the CH and the hospital. The Swedish ~~In another study~~ CH doctors reported that besides the differences in resources mentioned by the interviewed doctors in this article, the clinical experience and skill of the individual CH doctor could also influence which patients would be admitted (16).

Page 24:

CH doctors have previously described having to continuously assess risk, among other things manage the use of limited ambulance resources (16,32). An issue often brought up also by the participants in our study, with them arguing for keeping both technological and human resources close to the patient to reduce the need for transportation. ~~To decrease unnecessary ambulance use, participants of this study argued for keeping both technological and human resources close to patients, consistent with previously described continuous risk assessment by CH doctors (16,32).~~

Page 25:

An issue reported earlier by both CH doctors and managers from the same studied context includes the lack of IT support and insufficient adaptation of digital solutions (14, 16).

Page 26:

Many participants described the CHs as having cheaper inpatient beds compared to the general hospital, a notion shared with the doctors at the Swedish CHs (16), but not yet verified in any Swedish cost-effectiveness study.

VERSION 2 - REVIEW

**Reviewer**	**1**
**Name**	**Schmiedhofer, Martina**
**Affiliation**	**Charité Universitätsmedizin Berlin, Emergency and Acute Medicine**
**Date**	**25-Sep-2026**
**COI**	

The authors have addressed the main requests from the review report. The reviewer is also convinced by the replies to the other issues.
